# Caregiving Network Characteristics and Mental Health Care Utilization by Older Adults

**DOI:** 10.1111/jgs.70082

**Published:** 2025-09-04

**Authors:** Mary F. Wyman, Josephine Jacobs, Lily Stalter, Manasa Venkatesh, Ranak B. Trivedi, Amy L. Byers

**Affiliations:** 1 W.S. Middleton Memorial Veterans Hospital, Madison, Wisconsin, USA; 2 Department of Medicine, Division of Geriatrics, University of Wisconsin School of Medicine & Public Health, Madison, Wisconsin, USA; 3 Department of Psychiatry, University of Wisconsin School of Medicine & Public Health, Madison, Wisconsin, USA; 4 Health Economics Resource Center, VA Palo Alto Health Care System, Menlo Park, California, USA; 5 Department of Health Policy, Stanford University School of Medicine, Stanford, California, USA; 6 Department of Surgery, University of Wisconsin, Madison, Wisconsin, USA; 7 HSR&D Center for Innovation to Implementation, Palo Alto VA Medical Center, Palo Alto, California, USA; 8 Department of Psychiatry and Behavioral Sciences, Stanford University, Palo Alto, California, USA; 9 Department of Psychiatry and Behavioral Sciences; Division of Geriatrics, Department of Medicine, University of California, San Francisco, California, USA; 10 Center for Data to Discovery and Delivery Innovation, San Francisco VA Health Care System, San Francisco, California, USA

**Keywords:** dementia, mental health, veteranscaregiving

## Abstract

**Background:**

Rates of mental health service use are low among older adults. This study examined associations between mental health care utilization and caregiving network characteristics, including caregiving network size, caregiving intensity, the presence of formal helpers, and primary caregiver characteristics.

**Methods:**

Using a sample of 692 respondents in the health and retirement study (HRS) with linkage to veterans affairs healthcare records (mean age = 78.7, SD = 8.08; 97.1% male), logistic regression models tested caregiving network characteristics as predictors of mental health care utilization, adjusting for demographics and health conditions. Moderation effects of cognitive status (Langa-Weir HRS Classification) and depressive symptoms (CESD-8 Scale) were explored.

**Results:**

Mean network size was 1.6 helpers (SD 1.0), with 77.6% of networks comprising only family caregivers and 41.7% of care recipients reporting high-intensity caregiving. Primary caregiver was most often a spouse (61.1%) and female (89.4%, *n* = 49 (7.1%) missing data). In multivariable models, primary caregiver female gender was associated with a three-fold increase in likelihood of mental health service utilization (OR = 3.95, 95% CI 1.72–9.05), while other caregiving network characteristics were not associated. Having a primary caregiver who was female was most strongly associated with service utilization for care recipients with less severe depressive symptoms or impaired cognition.

**Conclusions:**

Caregiver characteristics, particularly gender, may be important factors in facilitating greater mental health care use for older veterans. Targeted education and support may improve caregivers’ effectiveness as a resource to help reduce age-related disparities in mental health access. This study contributes to the growing body of research examining the impact of caregivers on healthcare outcomes of older adults.

## 1 | Introduction

Mental disorders are common and cause a high burden and disability in late life [1], and mental health (MH) services such as diagnostic assessments, individual or group psychotherapy, and psychiatric medications can serve to reduce symptoms of mental illness and substance use disorders. Yet MH service utilization rates are low among older adults [2]. Studies have identified intrinsic factors impacting utilization, such as concerns about stigma and negative beliefs about MH care [3, 4], as well as extrinsic barriers to help-seeking (e.g., logistic issues such as transportation) [5]. Despite decades of work in this area, the age-related treatment gap persists, with MH utilization rates among older adults hovering at one-third those of younger adults in both community [6] and Veterans Affairs (VA) [7] samples. Further examination of facilitators and barriers to MH use is urgently needed, especially in light of the aging of the global population [8].

A growing body of research focuses on the role of caregivers—family, friends, or professionals who provide assistance to older adults—in shaping healthcare use [9]. For example, larger caregiving network size has been linked to receiving end-of-life care consistent with patient preference [10] and to being accompanied at physician visits [11]. Moreover, the care type “mix”—the balance of family caregivers versus formal/professional helpers in the network—is increasingly recognized as an important focus of research to guide health policy [12]. Previous work by our group demonstrated that receipt of any caregiving is associated with a greater likelihood of MH use [13]. However, little is known about the importance of specific caregiving network characteristics for MH utilization. Moreover, while there is a robust literature documenting differences by caregiver relationship status and gender [14, 15], including in caregivers’ utilization of supportive resources [16], the role of these characteristics in care recipient healthcare utilization has rarely been examined. These gaps have particular relevance for military veterans, who are at increased risk for MH problems [17] but have well-documented low rates of MH treatment [18], on par with civilians in comparative studies [19].

### 1.1 | The Present Study

This study leveraged a unique data resource combining nationally representative survey data with VA healthcare records to identify caregiver characteristics that are associated with MH utilization by the care recipient. We hypothesized that larger network size and caregiving intensity, as well as having formal help in the caregiving network, would be associated with a higher likelihood of utilization. In addition, given prior work showing that MH service acceptance or use is higher among younger generations [20] and women [21], we expected that having a primary caregiver who is an adult child or grandchild, or who is female, would be related to a greater likelihood of MH use. Finally, we sought to explore moderation effects of care recipient cognitive status and depression severity.

## 2 | Methods

### 2.1 | Sample

The sample was derived from a cohort of Health and Retirement Study (HRS) respondents who self-identified as military veterans, consented to linkage with VA records, and could be successfully matched [22]. The HRS is a nationally representative survey of the US population aged > 50, with comprehensive data collection every 2 years and ongoing sample replenishment. For each linked veteran, a dataset of VA healthcare records from 1999 to 2013 was created.

At each wave, HRS respondents report on the receipt of caregiver assistance in the last month related to functional deficits expected to last ≥ 3 months. For help with activities of daily living (ADLs), instrumental ADLs (IADLs), and financial management, details are gathered on who helped (up to 15 individuals, in addition to professional care workers) and for how many hours. For this study, linked veterans who (1) participated in HRS waves 5 (2000) through 11 (2012) and (2) received caregiving during this timeframe were eligible. Respondents were excluded if they were living in a residential facility or if they were < 60 years old. The study tested cross-sectional associations, using a dynamic baseline with one datapoint per respondent taken from the first wave between 2000 and 2012 in which they reported caregiving receipt (*N* = 692). The study was approved by the local Institutional Review Board.

### 2.2 | Outcome/Dependent Variable: Utilization of Outpatient MH Services

We created our binary outcome using all available information on MH utilization, an approach documented in detail elsewhere [13]. Briefly, we first counted an affirmative answer to either of two HRS items on self-reported (a) psychiatric or psychological treatment and (b) medication use for psychiatric symptoms. Second, use of VA-based specialty MH care was calculated using a care episode categorization system [23], counting ≥ 1 encounter in the Psychiatry or Substance Abuse categories. Third, because older adults commonly seek MH help in the primary care setting [24], we counted ≥ 1 primary care encounter with a linked MH diagnosis (Table S1), consistent with previous work [17, 25]. We counted VA utilization in a 24-month timeframe starting at the HRS interview date.

### 2.3 | Key Predictors: Caregiving Network Characteristics

Caregiver characteristics were measured at the care recipient level and derived from HRS data [26].

1. *Caregiving network size* captures the total number of helpers for ADL, IADLs, and financial management tasks over the past month.
2. *Caregiving frequency* reflects total days in the past month on which help was received, across all helpers (max. 31 days).
3. *Caregiving amount* reflects total hours of caregiving received in the past month, across all helpers (max. 744 h). For analysis, we created a binary variable to identify *high-intensity caregiving receipt* of ≥ 20 h per week (≥ 80 vs. < 80 h per month) [27].
4. *Care type mix* [12] was dichotomized into respondents with any formal helpers versus only family helpers in their caregiving network as per self-report in the HRS interview. Helpers reported by the care recipient to be affiliated with an “organization” or as an “employee” of an institution were classified as *formal*. All other helpers, including friends and other non-relatives, were classified as *family*.
  Primary caregiver (CG) was defined as the helper with the most frequent contact (if there were multiple individuals helping the same number of days, this was the helper providing the greatest amount of care, i.e., the most hours).
5. *Primary CG relationship status* was categorized as spouse/partner; child/grandchild-level (including child-in-law, grandchild-in-law, and stepchild); or other (sibling, sibling-in-law, other relative, or non-related individuals, including formal helpers), consistent with previous work [16].
6. *Primary CG gender* was categorized as male vs. female (non-binary options were not queried) using HRS variables capturing respondent-reported helper’s sex and spouse’s sex when the spouse provided care. If needed, we extrapolated gender from the relationship (e.g., daughters, sisters, and mothers were assumed female; sons, brothers, and fathers were assumed male). The gender of formal helpers was not assessed.

### 2.4 | Potential Confounders: Care Recipient Characteristics

We adjusted models with care recipient characteristics derived from the HRS. *Demographics* included age in years, gender (male or female) and race and ethnicity (categorized as non-Hispanic White, non-Hispanic Black, or Hispanic/Other; “other” is an HRS category used to preserve respondent privacy and includes American Indian, Alaskan Native, Asian, Native Hawaiian, or Pacific Islander self-reported race.) *Physical health status* was assessed with the count of 7 medical conditions (high blood pressure, diabetes, cancer, lung disease, heart disease, stroke, and arthritis). *Educational attainment* was categorized in 4 levels: < high school (HS) diploma, HS or General Educational Development (GED) completion, some college, or ≥ 4-year college degree.

### 2.5 | Moderators: Care Recipient Cognitive Status and Depressive Severity

*Care recipient cognitive status* was dichotomized as normal versus impaired using the Langa-Weir classification which uses objective assessments conducted on HRS participants [28]. *Depressive severity* in the past week was measured with the 8-item Center for Epidemiological Studies-Depression scale CESD-8 [29]; and dichotomized using the previously validated cutpoint of ≥ 3 [30].

### 2.6 | Statistical Analysis

#### 2.6.1 | Main Analyses

First, we characterized the sample using means for continuous variables and percentages for categorical variables, and t-tests and Chi-square tests to compare care recipients with and without MH utilization. We then conducted logistic regression analyses examining individual caregiving network characteristics and their ability to predict utilization. To assess independent contributions of potential predictors, we used multivariable logistic regression with all caregiving network characteristics in the model and adjusting for potential confounders listed above (care recipient demographics, physical health status, and educational attainment). Available HRS weights are based on wave-specific characteristics of the nationally representative sample to allow inference to the US population over age 50 and account for oversampling of certain households. Because the weights do not infer directly to this subsample of veterans, we conducted unweighted model analyses and instead adjusted for care recipient age and race/ethnicity (included among the characteristics used to construct sampling weights that account for differential selection at baseline), as per previously validated recommendations [31, 32].

#### 2.6.2 | Supplemental and Moderation Analyses

In supplemental, exploratory analyses, we first undertook a deeper examination of caregiving characteristics that emerged as significant in the primary models with descriptive statistics and subgroup comparisons. Next, we examined whether care recipient cognitive status and depression severity modified the associations between caregiving network characteristics and care recipient MH services utilization. Following primary analyses, we explored interaction effects between each of these variables and caregiving factors using established approaches [33, 34], setting the significance threshold a priori at *p* < 0.2 to justify subsequent subgroup analyses.

All analyses were performed using SAS software (version 9.4, SAS Institute; Cary, North Carolina, U.S.) and significance was set at the *p* < 0.05 level.

## 3 | Results

### 3.1 | Sample Descriptives (Table 1)

#### 3.1.1 | Care Recipient Characteristics

Care recipients had an average age of 78.7 (SD = 8.08), and 97.1% were male, with most (66.2%) having an education level of HS/GED diploma or less. The majority were non-Hispanic White (78.3%; *n* = 542) and were partnered or married (71.4%). A mean of 3.2 (SD = 1.5) health conditions was reported, with 65.0% of the sample being classified with impaired cognition and 27.5% reporting significant depressive symptoms. Of the entire sample, *n* = 195 (28.2%) reported MH service use. Care recipients with MH utilization tended to be younger, were more likely to be married, and reported more depressive symptomatology.

#### 3.1.2 | Caregiving Network Characteristics

Caregiving network size ranged from 1 to 7 with an average size of 1.6 (SD = 1.0); 416 (60.1%) had one helper and 104 (15.0%) had ≥ 3 helpers. Received caregiving frequency averaged 20.7 days/month (SD = 12.7) and received caregiving amount averaged 169.6 h/month (SD = 239.6). Receipt of high-intensity caregiving was reported by 278 (41.7%) care recipients. Most networks comprised family caregivers only (77.8%), with the remainder (22.2%) including ≥ 1 formal helper. Most primary CG were female (89.4%, *n* = 49 (7.1%) missing data) and a spouse (61.1%).

### 3.2 | Modeling Mental Health Services Utilization

Univariable logistic regression models revealed that of the five caregiving network factors, only primary CG gender was associated with utilization (OR = 2.56 (95% CI 1.28, 5.12), *p* = 0.008; results not shown). Partially adjusted models accounting for care recipient race/ethnicity and age produced similar results (OR = 2.43, 95% CI 1.20–4.88; see Table 2). In the full multivariable model, primary CG gender retained a statistically significant association (OR = 3.95, 95% CI 1.72–9.05).

In supplemental analyses, we further explored primary CG gender by caregiving characteristics and MH services utilization. Female primary CGs were more likely to be the sole caregiver (*N*_female_ = 364/575 (63.3%) vs. *N*_male_ = 20/68 (29.4%), *p* < 0.0001). As seen in Table 3, female CGs were typically spouses (*n* = 418; 72.7%) while male CGs were most likely at the child- or grandchild-level (*n* = 46; 67.7%). Care recipients with female primary CGs were less likely to have formal helpers in the network (18.2% vs. 29.4%, *p* = 0.03). There were no significant differences in frequency or amount of care received.

### 3.3 | Moderation Analyses: Care Recipient Cognitive Status and Depressive Severity

**FIGURE 1 |:**
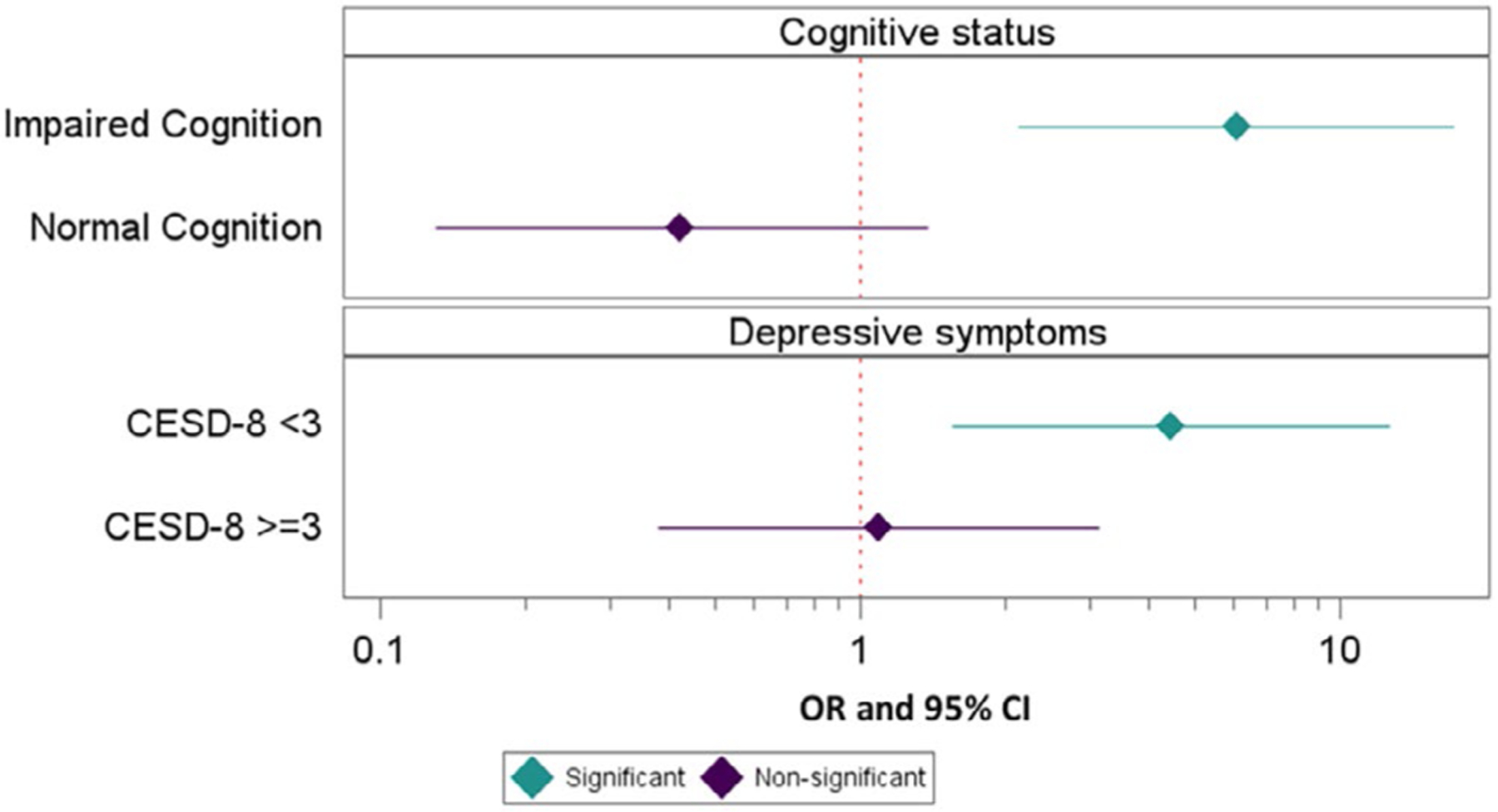
Primary caregiver gender associated with mental health care utilization among subgroups of cognition and CESD-8 depression score. Multivariable logistic regression predicting any mental health services utilization by care recipient; OR and 95% CI for female vs. male gender. CESD-8 cutpoint indicates no or minimal depressive symptoms (< 3) versus significant depressive symptoms (≥ 3). All models adjusted for care recipient age and race/ethnicity. Sample includes care recipients with family caregivers with available gender data; normal cognition model *n* = 231, impaired cognition model *n* = 412, CESD-8 < 3 model *n* = 463, and CESD-8 ≥ 3 model *n* = 180. *Note:* X-axis is shown on a log scale for ease of display.

An interaction effect for cognitive status × PCG gender was significant at the 0.2 level (*b* = 0.675, 95% CI 0.29–1.06, SE = 0.20, *p* = 0.0006), justifying subgroup analyses. In models adjusted for care recipient age and race (due to low subgroup numbers, other covariates were not included), cognitive status moderated the association of PCG gender and utilization, such that care recipients classified as having impaired cognition were more likely to use services if the PCG was female (OR = 6.08, 95% CI 2.14–17.28). In the normal cognition group, a converse effect emerged, but this was non-significant (OR = 0.424, 95% CI 0.13–1.38; see Figure 1).

For depression severity as measured by CESD-8 score, an interaction effect with PCG gender also was significant at the 0.2 level (*b* = −0.364, 95% CI −0.73–0.00, SE = 0.19, *p* = 0.052). For care recipients with no or mild depressive symptoms, PCG female gender was associated with a 3-fold increase in utilization (OR = 4.45, 95% CI 1.56–12.69). There was no significant association for care recipients with more severe depressive symptoms (CESD-8 ≥ 3 pts; OR = 1.09, 95% CI 0.38–3.14; see Figure 1).

## 4 | Discussion

In this national sample of older, predominantly male veterans receiving caregiving, most caregiver characteristics—including network size, caregiving intensity, presence of formal help, and relationship status—were not associated with MH utilization. Only having a primary caregiver who was female was related to increased likelihood of MH service use, and then only for care recipients with impaired cognition or no to mild depressive symptoms.

Previous research has identified gender differences among caregivers in their psychiatric symptoms, caregiver burden, and coping strategies [35–37], including those assisting persons with mental illness [38]. Although findings on helpers of older adults have been mixed [39], female caregiver gender predicted greater utilization of MH services by children and adolescents [40]. Our findings add to this literature, providing some of the first evidence for a link between caregiver gender and MH service utilization by the older care recipient. This association may be driven by gender differences in how emotional distress and MH services are viewed. Compared to male caregivers, women may “care more about”, in addition to “caring for”, the care recipient [41], leading to greater attunement to care recipient well-being [36]. Consistent with past work [37], we found that female primary CG provided more frequent assistance than their male counterparts, perhaps facilitating this attunement. Older men confide primarily in (typically female) spouses, and family members’ own experiences with MH care can influence help-seeking [42]. Gender differences in help-seeking for MH concerns have been well-documented, with women showing greater openness [21, 43]. Research on adolescent MH service use—another population for which caregiver facilitation of help-seeking can be necessary—shows that caregiver attitudes toward help-seeking and MH stigma are primary predictors of MH utilization [44, 45].

This converging evidence fits with moderation analyses results. Female caregivers may be more likely to identify MH concerns and pursue help. It may be that the treatment needs of older adults with more severe and intrusive symptoms are likely to be identified by themselves, caregivers, or health care providers. Older care recipients with mild symptoms, which may be less obvious and less disabling, and therefore more difficult to detect, benefit more from the presence of female helpers. Similarly, for care recipients with impaired cognition and thus greater dependence on caregivers for treatment decision-making and facilitation, caregiver attunement and openness might be important facilitators of MH service use.

The lack of associations with the other caregiving characteristics is in line with past research, including findings from a nationally representative sample of older adults with dementia, for whom caregiving network size was not related to healthcare utilization [11]. We found that primary CG relationship status showed no association, an unexpected result given that age variations in attitudes toward MH service use are well documented, including during our study timeframe [20]. It may be that our relationship status variable did not capture relevant cohort differences. On the other hand, our findings may underline the limits of the influence of spousal caregivers, as well as child/grandchild and other caregivers, to influence the care recipient’s use of MH services. Taken together, the evidence on the impact of caregiving characteristics on MH utilization is sparse and mixed [12], suggesting it is a potentially significant yet complex entity in need of further examination. More research is needed to identify which attributes are important and could be amenable to intervention.

Our findings are highly relevant to MH service delivery for older adults. Anticipated future shortages of the availability of formal and family caregivers, and recent calls for inclusive care [46], require a more nuanced understanding of how caregivers interface with the health care system and the older adult [47]. Older adults with mental illness often require in-home care [48], and these caregiving networks are a potentially powerful resource to increase the use of high-value care in later life. Despite evidence of gradually increasing use of both VA and non-VA MH services by veterans [13, 17], 17, the identification of intervention targets to increase appropriate MH utilization remains critical. Our results underscore the importance of tailored trainings and suggest that male caregivers may particularly benefit from education related to MH and encouragement to facilitate appropriate MH service use by the care recipient.

### 4.1 | Strengths and Limitations

A major strength of this study is the unique linkage of rich survey and healthcare records data, leveraging a novel resource for our exploration of caregiver-related factors in MH use for older adults. A limitation is our inability to include factors that may influence access to MH care in our models—for example, the local health care market, employer- or government-provided health insurance, and eligibility for VA healthcare services—due to the complexities of the linked dataset and the long study timeframe. Work to clarify the role of these factors in influencing MH care use by older adults, especially by Veterans and their caregivers, remains a critical future direction. Further, we did not have data that allowed us to account for mental health conditions in the model beyond the exploration of CESD-8 score as a moderator. While the study timeframe did not allow inclusion of recent changes in MH service delivery (e.g., emerging from COVID-19 pandemic), the caregiving landscape has remained relatively consistent over the past decades, apart from modest growth in formal in-home assistance [49, 50]. Moreover, previous work by our group demonstrated the sustained importance of caregivers for MH service use across time and after a system-wide VA MH expansion [13]. This provides reassurance regarding our findings, though future work examining the role of caregivers should include such access and time-based factors. Generalizability may be limited due to the unique aspects of veteran health and aging, and future work should examine parallels between caregiving for veterans and non-veterans. Further, while most primary caregivers were female, consistent with other studies [36], care recipients were predominantly male, which may have influenced our findings in unknown ways. Nevertheless, the large proportion of male helpers who are not spouses is a strength of the study, as these caregivers are often underrepresented in research. Missing information for CG gender meant that some family and all formal caregivers were excluded from those analyses. Finally, given the limitations of the dataset, we were limited in our ability to compare the effects of formal vs. family caregivers, nor could we account for caregivers’ existing knowledge level, attitudes toward the care recipient’s MH symptoms [44], or past experience with MH services, all of which may be important factors influencing help-seeking behavior. Future studies should address these and additional modifiable characteristics of caregivers, such as caregiver strain [40]. These issues are highly relevant to informing the development of effective interventions for caregivers and geriatric workforce enhancement programs, and should continue to be a focus of research.

## 5 | Conclusions

In conclusion, this study adds to the growing body of research examining caregiving characteristics and healthcare utilization, a critical area of focus in light of growing acknowledgment of the vital importance of caregivers in facilitating healthcare for older adults. Building on the current study, future research can help inform the development of caregiver-centered care interventions to support appropriate MH service use among older adults who can benefit.

## Supplementary Material

Suppl Table S1

Additional supporting information can be found online in the Supporting Information section. Table S1: ICD-9 diagnoses and VA clinic locations (stop codes) used to determine primary care-based mental health utilization.

## Figures and Tables

**TABLE 1 |:** Characteristics^a^ of sample, stratified by mental health services utilization in same wave.

		Entire sample *N* = 692		Had MH service use *N* = 195		No MH service use *N* = 497		Degrees of freedom	*p* ^b^
		*N*	%^c^	*N*	%	*N*	%	df	*p*
Care recipient characteristics									
Age, mean ± SD		78.70 ± 8.08		76.84 ± 7.93		79.43 ± 8.03		690	< 0.0001
Age group	60–69	104	15.03%	41	21.03%	63	12.68%	3	0.003
	70–79	244	35.26%	73	37.44%	171	34.41%		
	80–89	294	42.49%	75	38.46%	219	44.06%		
	90+	50	7.23%	6	3.08%	44	8.85%		
Gender^d^	Male	672	97.11%	188	96.41%	484	97.38%	1	0.491
Race and ethnicity^d^	White non-Hispanic	542	78.32%	151	77.44%	391	78.67%	2	0.912
	Black non-Hispanic	101	14.60%	29	14.87%	72	14.49%		
	Other/Hispanic	49	7.08%	15	7.69%	34	6.84%		
Partnered status	Married or partnered	493	71.35%	150	76.92%	343	69.15%	1	0.042
Education^d^	< High school	216	31.21%	55	28.21%	161	32.39%	3	0.076
	HS grad/GED	242	34.97%	60	30.77%	182	36.62%		
	Some college	139	20.09%	50	25.64%	89	17.91%		
	≥ College degree	95	13.73%	30	15.38%	65	13.08%		
Number of health conditions^e^, mean ± SD		3.24 ± 1.47		3.41 ± 1.54		3.18 ± 1.44		690	0.063
Used proxy report		225	32.51%	70	35.90%	155	31.19%	1	0.234
Center for Epidemiologic	0–2 pts	502	72.54%	129	66.15%	373	75.05%	1	0.018
Studies Depression Scale—8 items (CESD-8) score	3–8 pts	190	27.46%	66	33.85%	124	24.95%		
Cognitive status^f^	Normal	242	34.97%	67	34.36%	175	35.21%	1	0.833
	Impaired	450	65.03%	128	65.64%	322	64.79%		
Caregiving network characteristics									
Caregiving network size, number of helpers, mean ± SD		1.64 ± 1.00		1.63 ± 0.98		1.65 ± 1.01		690	0.859
Caregiving frequency^g^, days per month, mean ± SD		20.74 ± 12.65		21.01 ± 12.63		20.63 ± 12.67		687	0.729
Caregiving amount^h^, hours per month, mean ± SD		169.61 ± 239.61		176.22 ± 249.61		167.04 ± 235.85		664	0.658
Caregiving intensity	Low (< 80 h/month)	388	58.26%	109	58.60%	279	58.13%	1	0.911
	High (≥ 80 h/month)	278	41.74%	77	41.40%	201	41.88%		
Care type mix	Any formal help	154	22.25%	40	20.51%	114	22.94%	1	0.490
	Family help only	538	77.75%	155	79.49%	383	77.06%		
Primary caregiver relationship	Spouse/partner^i^	423	61.13%	128	65.64%	295	59.36%	2	0.242
	Child/grandchild	147	21.24%	34	17.44%	113	22.74%		
	Other	122	17.63%	33	16.92%	89	17.91%		
Primary caregiver gender^j^	Female	575	89.42%	176	94.62%	399	87.31%	1	0.006
Utilization of mental health services									
Mental health services utilization	HRS self-report^k^	119	18.71%	119	67.23%	—			—
	VA specialty MH care services	65	9.39%	65	33.33%	—			—
	VA Primary care-based MH services	83	11.99%	83	42.56%	—			—

a Measured at the first HRS wave, they reported caregiving receipt. MH utilization occurred within that same wave measurement period.

b *T*-tests were used for continuous variables and Chi-squared for categorical variables.

c Column percentages are reported throughout table.

d Gender, race/ethnicity, and education level are measured at baseline or per HRS RAND protocol. Non-binary gender identity was not queried. “Other” race category is a feature of HRS data collection to preserve participant privacy and include self-identified American Indian, Alaskan Native, Asian, Native Hawaiian, and Pacific Islander race.

e Possible scores range from 0 to 7. The seven included health conditions are: high blood pressure, diabetes, cancer, lung disease, heart disease, stroke, and arthritis.

f Cognitive status classification used the Langa-Weir algorithmic approach, which uses objective cognitive assessment data from the HRS 28.

g Caregiving frequency *N* = 689 (missing *n* = 3, 0.04%).

h Caregiving amount and caregiving intensity *N* = 688 (missing *n* = 26, 3.8%).

i “Spouse/partner” includes married or unmarried domestic partner. “Child/grandchild” includes children, grandchildren, and in-laws at these levels. “Other” includes siblings, extended family, friends, neighbors, and formal helpers.

j Primary caregiver gender *N* = 643 (missing *n* = 49, 7.1%).

k HRS self-report *N* = 636 (missing *n* = 56, 8.1%).

**TABLE 2 |:** Logistic regression models examining associations of mental health services utilization with caregiving network and care recipient factors.

		Individual factors^a^		Full multivariable model^a,b^	
		OR	95% CI	OR	95% CI
Caregiving network characteristics					
Caregiving network size		1.04	0.87–1.23	1.03	0.83–1.28
Caregiving amount	Low-intensity (< 80 h/month) High-intensity (≥ 80 h/month)	Ref 1.07	0.75–1.52	Ref 1.04	0.70–1.54
Care type mix	Any formal help in network^c^	1.02	0.67–1.54	1.09	0.64–1.85
Primary caregiver relationship	Spouse/partner^d^	Ref	0.52–1.29	Ref	0.74–2.11
	Child/grandchild	0.82	0.58–1.46	1.25	0.79–2.50
	Other	0.92		1.40	
Primary caregiver gender	Men Women	Ref **2.43**	1.20–4.88	Ref **3.95**	1.72–9.05
Care recipient characteristics					
Gender	Men/other Women	Ref 1.47	0.57–3.81	Ref 1.79	0.54–5.93
Education	< High school High school grad/GED Some college College and above	Ref 0.88 1.46 1.29	0.57–1.36 0.91–2.35 0.75–2.24	Ref 1.03 1.41 1.27	0.65–1.64 0.84–2.35 0.69–2.32
Number of health conditions		1.11	0.99–1.24	1.08	0.96–1.23

a All models also include care recipient age and race/ethnicity (White non-Hispanic, Black non-Hispanic, and Other/Hispanic) in lieu of weighting [31]. Significant ORs are bolded (*p* < 0.05; 95% CI).

b Full multivariable model includes all factors in one model (*N* = 619). Missingness imposed by primary CG gender (*n* = 49 missing) and caregiving intensity (*n* = 26 missing). Two participants were missing both variables.

c Any formal help refers to networks with a caregiving type mix that includes only formal (professional) helpers (*N* = 34 recipients) or both formal and family caregivers, independent of what primary caregiver type is.

d “Spouse/partner” includes married or unmarried domestic partner. “Child/grandchild” includes children, grandchildren, and in-laws at these levels. “Other” includes siblings, extended family, friends, neighbors, and formal helpers.

**TABLE 3 |:** Caregiving characteristics and mental health services utilization by primary caregiver gender (*N* = 643).

	Female *N* = 575		Male *N* = 68		Degrees of freedom	*p* ^a^
	*N*	%	*N*	%	df	*p*
Caregiving characteristics						
Caregiving network size, mean ± SD	1.59 ± 0.98		2.22 ± 1.16		78.7	< 0.0001
Caregiving frequency^b^, days per month, mean ± SD	21.55 ± 12.27		18.87 ± 13.23		639	0.094
Caregiving amount^c^, hours per month, mean ± SD	179.89 ± 245.21		149.41 ± 219.46		617	0.335
High intensity caregiving received (≥ 80 h/month)	239	43.22%	28	42.42%	1	0.902
Care type mix: Any formal help	105	18.26%	20	29.41%	1	0.028
Relationship to CR	418	72.70%	5	7.35%	2	< 0.0001
Spouse/partner	91	15.83%	46	67.65%		
Child/grandchild	66	11.48%	17	25.00%		
Other						
MH utilization						
MH use in wave, any type	176	30.61%	10	14.71%	1	0.0006
HRS self-report^d^	104	19.70%	8	12.90%	1	0.197
VA specialty MH care services	62	10.78%	2	2.94%	1	0.041
VA Primary care-based MH services	78	13.57%	3	4.41%	1	0.032

a *T*-tests were used for continuous variables and Chi-squared for categorical variables. Column percentages are shown.

b Caregiving frequency *N* = 641.

c Caregiving amount and caregiving intensity *N* = 619.

d HRS self-report *N* = 590.

## Summary

- Key points
  - Mental health service use is lower among older adults compared to younger adults, and caregivers may have a role in facilitating access for those who need help.
  - In a sample of older veterans receiving caregiving, we found that having a primary caregiver who was female was strongly associated with mental health service utilization (odds ratio 3.95).
  - This association was strongest for care recipients who had less severe depressive symptoms or impaired cognition.
- Why does this paper matter?
  - Findings suggest that caregiver characteristics may impact mental health care utilization by older adults.
  - Targeted education and support may improve caregivers’ effectiveness as a resource to help reduce age-related disparities in mental health service utilization.
