# Supplementary material for: Caregiving Network Characteristics and Mental Health Care Utilization by Older Adults
Source: J Am Geriatr Soc. 2025 Sep 4;73(11):3376–86. doi: 10.1111/jgs.70082 (PMC12584981; doi:10.1111/jgs.70082)
Supplement: Supplementary file 1 — Table S1: ICD‐9 diagnoses and VA clinic locations (stop codes) used to determine primary care‐based mental health utilization. [file JGS-73-3376-s001.pdf]

Supplemental Table S1. ICD-9 diagnoses and Veterans Affairs clinic locations (stop codes) used to determine primary care-based mental health utilization.

Mental Health Disorder Category	Description	ICD-9 Code
**Mood Disorders**	Major Depression, single episode	29620 29621 29622 29623 29624 29625 29626
	Major Depression, recurrent	29630 29631 29632 29633 29634 29635 29636
	Dysthymia	3004
	Bipolar disorder	29600 29601 29602 29603 29604 29605 29606 29610 29611 29612 29613 29614 29615 29616 29640 29641 29642 29643 29644 29645 29646 29650 29651 29652 29653 29654 29655 29656 29660 29661 29662 29663 29664 29665 29666 2967 29680 29681 29682 29689
	Mood disorder due to known physiological condition or unspecified	29383, 29690 29699 311
**Anxiety Disorders/ PTSD**	Post-traumatic stress disorder	30981
	Generalized anxiety disorder	30002
	Panic disorder	30001 30021
	Phobia	30000 30010 30011 30012 30013 30014 30015 30016 30019 30020 30022 30023 30029 3003 3070 30720 30721 30722 30723 3073
	Somatoform disorders	3005 3006 3007 30081 30082 30089
**Substance Use Disorders**	Alcohol use disorders	V6542 30300 30301 30302 30303 30390 30391 30392 30393 30500 30501 30502 30503
	Nicotine use disorders	V1582 3051
	Drug use disorders	30400 30401 30402 30403 30410 30411 30412 30413 30420 30421 30422 30423 30430 30431 30432 30433 30440 30441 30442 30443 30450 30451 30452 30453 30460 30461 30462 30463 30470 30471 30472 30473 30480 30481 30482 30483 30490 30491 30492 30493 30520 30521 30522 30523 30530 30531 30532 30533

		30540 30541 30542 30543<br>30550 30551 30552 30553<br>30560 30561 30562 30563<br>30570 30571 30572 30573<br>30580 30581 30582 30583<br>30590 30591 30592 30593
**Sleep Disorders**	Insomnia, Circadian rhythm sleep disorder, etc.	32702 78052 30740 30741<br>30742 30743 30744 30745<br>30746 30747 30748 30749
**Other Disorders**	Unspecified psychosis not due to a substance or known physiological condition	2970 2971 2972 2973 2978<br>2979 2980 2981 2982 2983<br>2984 2988 2989
	Schizophrenia	29500 29501 29502 29503<br>29504 29505 29510 29511<br>29512 29513 29514 29515<br>29520 29521 29522 29523<br>29524 29525 29530 29531<br>29532 29533 29534 29535<br>29540 29541 29542 29543<br>29544 29545 29550 29551<br>29552 29553 29554 29555<br>29560 29561 29562 29563<br>29564 29565 29570 29571<br>29572 29573 29574 29575<br>29580 29581 29582 29583<br>29584 29585 29590 29591<br>29592 29593 29594 29595
	Personality disorder	3010 30110 30111 30112<br>30113 30120 30121 30122<br>3013 3014 30150 30151 3016<br>3017 30181 30182 30183<br>30184 30189 3019
	Adjustment disorder	3080 3082 3083 3084 3089<br>3090 30924 30928 30929<br>3093 3094
	Sexual disorder	3020 3021 3022 3023 3024<br>30250 30251 30252 30253<br>3026 30270 30271 30272<br>30273 30274 30275 30276<br>30279 30281 30282 30283<br>30284 30285 30289 3029

Veterans Affairs clinic locations (stop codes): 323 (primary care), 326 (geriatrics/telephone), 338 (primary care/telephone), 350 (geriatric primary care), 170 (home based primary care (HBPC)/physician), 172 (HBPC non-physician prescribing provider; NP, CNS, or PA), 178 (HBPC/telephone), 534 (MH integrated care/individual), 537 (psychosocial rehabilitation/telephone)
